# Fighting the ‘Infodemic’: Legal Responses to COVID-19 Disinformation

**DOI:** 10.1177/2056305120948190

**Published:** 2020-07-30

**Authors:** Roxana Radu

**Affiliations:** 1 University of Oxford, UK; 2 University of Geneva, Switzerland

**Keywords:** COVID-19, access to information, legislation, online disinformation, online trust

## Abstract

Online disinformation has been on the rise in recent years. A digital outbreak of disinformation has spread around the COVID-19 pandemic, often referred to as an “infodemic.” Since January 2020, digital media have been both the culprits of and antidotes to misinformation. The first months of the pandemic have shown that countering disinformation online has become as important as ensuring much needed medical equipment and supplies for health workers. For many governments around the world, priority COVID-19 actions included measures such as (a) providing guidance to social media companies on taking down contentious pandemic content (e.g., India); (b) establishing special units to combat disinformation (e.g., EU, UK); and (c) criminalizing malicious coronavirus falsehood, including in relation to public health measures. This article explores the short and potential long-term effects of newly passed legislation in various countries directly targeting COVID-19 disinformation on the media, whether traditional or digital. The early actions enacted under the state-of-emergency carve new directions in negotiating the delicate balance between freedom of expression and online censorship, in particular by imposing limitations on access to information and inducing self-restraint in reporting. Based on comparative legal analysis, this article provides a timely discussion of intended and unintended consequences of such legal responses to the “infodemic,” reflecting on a basic set of safeguards needed to preserve trust in online information.

‘We’re not just fighting an epidemic; we’re fighting an infodemic. Fake news spreads faster and more easily than this virus, and is just as dangerous’ (World Health Organization [WHO], 2020). This concern, expressed by the WHO Director-General on 15 February, reiterated the danger posed by large-scale online disinformation in the context of the COVID-19 pandemic. Alongside infections with the new severe acute respiratory syndrome coronavirus 2 (SARS-CoV-2), false cures and faulty prevention methods have also gone viral, putting more lives at risk. Just like the virus outbreak, disinformation campaigns keep surfacing at higher echelons in recent months. Since January, we have learned that countering disinformation online is as important as ensuring much needed medical equipment and supplies for health workers.

Like the pandemic itself, the digital outbreak of disinformation is global. Yet the legal responses and temporary measures put forward so far show local solutions are only part of the answer. In the absence of global scale approaches, the national pursuits may infringe upon long-fought battles for civil liberties. While solutions tailored to the national and local contexts are much needed, they do not represent the holy grail of countering the global phenomenon of disinformation. The remedy for pervasive online falsehood—trust in science and verified sources—comes mediated through accurate reporting and informed public debates. *Au contraire*, limiting access to information in times of crisis will have long-term consequences on our digital interactions, which have become indispensable to the functioning of our political, economic, research, and educational systems.

Since the coronavirus outbreak started, digital media have been both the culprits of and antidotes to disinformation. For many governments around the world, priority COVID-19 actions included measures that reflected the heightened importance of combating fake news, such as (a) criminalizing malicious coronavirus falsehood (Hungary, South Africa), (b) establishing special units to combat disinformation (e.g., EU, UK), and (c) providing guidance to social media companies on taking down contentious pandemic content (e.g., India). To counter the “infodemic,” 18 governments have added counter-measures via decrees and emergency legislation. The dismissive “fake news” discourse promoted by many politicians—legitimizing an offensive against independent journalism since 2016—has framed the legal responses to the COVID-19 disinformation, further eroding trust in the watchdog function of the media.

Specific actions taken during the pandemic build on existing disinformation counter-measures around the world, many of which have not proven their effectiveness. My recent research has shown that anti-misinformation measures adopted by governments vary greatly on a soft-hard law continuum; when legislation is passed, the regime type (democratic, non-democratic) does not influence significantly its content with respect to freedom of expression limitations and long-term chilling effects (De Gregorio and Radu, 2019). However, democracies tend to have more counter-measures that are publicly deliberated (task forces, media literacy courses, etc.), whereas autocracies tend to pass new laws or extend existing provisions to cases of disinformation. Vague definitions of “public interest,” “public safety,” “falsehood” in online falsehood laws, and their overtly broad scope have sometimes been used as a political tool to stifle media freedom and criticism. On a global scale, according to the Committee for the Protection of Journalists, the number of journalists detained under fake news provisions tripled since 2016 (Committee to Protect Journalists [CPJ], 2020a).

Perhaps unsurprisingly given it was the epicenter of the pandemic in February and March, Europe had the highest incidence of new laws fighting COVID-19 disinformation (six jurisdictions), followed by the Americas (four jurisdictions), Asia and Pacific, Middle East and North Africa (with three jurisdictions each) and Africa with two jurisdictions (International Press Institute, 2020). While the West has, over the past two decades, come to expect media regulation to be enacted through policy written and approved by elected officials or by independent regulators (Irion & Radu, 2013), in non-democratic countries, ruling by decree on matters affecting the media is common. Under the exceptional circumstances imposed by the pandemic, the legislation enacted by decree has sometimes conflicted with constitutional values, including the commitment to a healthy public sphere. Various degrees of limitations on the media have thus been enforced, from the criminalization of broadly defined false reporting to restrictions placed on freedom of expression in relation to prevention measures, quarantine, timing, and potential treatment for COVID-19. In a few cases, hastily passed decrees affecting media freedom have been rapidly repelled (Serbia, Bulgaria). The most problematic features of emergency legislation are discussed below.

Imposing jailtime for coronavirus disinformation has been a common in several countries. Backed by the two-thirds majority of his party, the Hungarian Prime Minister Viktor Orbán obtained the power to rule by decree during an extended national state of emergency and passed a new law criminalizing “false” or “distorted” COVID-19 information, with penalties involving hefty fines and up to 5 years in prison. Covertly, this law quashes public scrutiny related to measures taken by the government to address the pandemic, as it uses broad provisions without time limits (Polyak, 2020). Similar provisions were passed in Bolivia, where the decree no. 4200 issued by the Añez interim government on 25 March contained the following article: “individuals who incite non-compliance with this decree or misinform or cause uncertainty to the population will be subject to criminal charges for crimes against public health” (Art. 13-2), with penalties in fines and jail time ranging from 1 to 10 years (CPJ, 2020b). In South Africa, under the Disaster Management Act 2002, section 11(5) made it an offense penalized by fine or imprisonment for 6 months (or both) to “publish a statement through any medium with the intention to deceive about a narrow range of information related to the transmission of the virus, personal infection status and government measures to address the pandemic.” Although the scope of application is much more restricted, there are mounting fears (AfricaNews, 2020) that such legislation impedes journalists from performing a key function, that of holding leaders accountable (Deuze, 2007).

The second set of measures affecting media reporting during the pandemic centers around who is authorized to inform the public about the health situation and the measures taken in connection with SARS-CoV-2. In Serbia, a governmental decree enforced the centralization of COVID-19 information and imposed sanctions for local institutions releasing information to the media without authorization from the capital. Amid public criticism and following an explicit request of the President of Serbia, Aleksandar Vučić, the order was repelled a few days later over concerns of censorship and abolition of media freedom. In India, the government invoked fears of fake news causing panic and broad unrest during the lockdown. Prime Minister Narendra Modi demanded that journalists publish only official information (Goel & Gettleman, 2020), while the Supreme Court was urged to **“**issue a direction that no electronic/print media/web portal or social media shall print/publish or telecast anything without first ascertaining the true factual position from the separate mechanism provided by the Central Government**”** (Times of India, 2020).

The media capacity to impart timely and accurate information also came under increased challenge in countries such as Romania and Moldova, where the emergency legislation has doubled, and respectively tripled, the time authorities have to respond to Freedom of Information requests (Media Azi, 2020). Among the deluge of disinformation in relation to the pandemic, actions such as these limit the extent to which journalists can provide the public a service that disambiguates truth from falsehood. When the expectation of real-time information at our fingertips is omnipresent, undue restrictions imposed on access to information may lower even further the public’s capacity to verify news for their accuracy.

These early actions enacted under a state of emergency carve new directions in negotiating the delicate balance between freedom of expression and censorship online, not least by involving intermediaries (Chenou & Radu, 2017). Guidance issued by governments to large social media platforms targeting disinformation on coronavirus generally requested immediate action to take down or disable false or malicious content hosted on their platforms. The advisory of the Indian Ministry of Electronic & Information Technology released on 20 March 2020 is a case in point here. To police the public sphere according to more stringent guidelines mandated by various governments or in line with their updated community guidelines, social media platforms rely extensively on artificial intelligence (AI) tools. Facebook reported that 88.8% of all the removals related to hate speech and disinformation in the last quarter was detected by AI (Facebook, 2020). Raising the threshold of automation in content moderation gives us insights into the future, as many of these temporary measures may turn into permanent features once tested during a crisis.

Regardless of the lower performance of detection algorithms in languages other than English, having any government as the single or preferred source of information on social media is not desirable, as it may push citizens to search for alternative sources on platforms that are not filtering contentious information. Despite their high traffic, platforms owned by American giants such as Facebook, Google, Microsoft, and Twitter do not cover the entirety of the globe and a few hours are sufficient for a hoax to go viral on encrypted messaging services. At the same time, intermediaries enforcing their own policies around health disinformation may pay little attention to protecting media freedom and pluralism. My previous research showed that reigning in disinformation on social platforms is part and parcel of broader internet governance transformations which started in 2016 (Radu, 2019). During the COVID-19 crisis, stronger national approaches emerged and technology corporations acquired increased power as intermediaries that screen and take down content, in many cases in automated ways. The stringent measures newly imposed by governments and platforms to combat disinformation impact the governance approaches they sought to promote for decades, when they were advocating for democratic dialogue and multi-stakeholder deliberation ahead of decision-making.

The uncertainty around the duration of the coronavirus crisis raises novel challenges for the media sector and for society more generally. With no end in sight to the virus spread and a looming economic recession that has already started to hit hard the media industry, the fragility of our independent reporting system is put to test all over the world. Also put to the test is the safety net provided by civil liberty protections in democratic countries. The risk of such legislation staying in place for much longer than originally intended is real, as was the case with the 2001 Patriot Act, which extended the surveillance powers of the U.S. government with little oversight and increased extra-territorial powers.

Since the start of the pandemic, national legislation meant to discourage the creation and spread of misinformation also served to create the conditions under which it is more likely for it to flourish by undermining legitimate journalism and eroding trust in institutions of authority. Limiting access to information, criminalizing critical and “unpatriotic” reporting, and imposing faster, AI-driven content takedowns on social platforms will negatively impact our ability to distinguish between malicious and truthful information. Controlling scrutiny while weakening the media system has always been a temptation for public authorities and corporations alike, but international conventions have set minimum safeguards against that. Suspending these protections at a time when we need them the most will be counter-productive.

The trial and error approach emerging in newer democracies, as well as the many restrictions imposed on freedom of expression in democratic countries, show that figuring out the rules during the pandemic often results in excessive sanctions and deliberate restrictions directly affecting the role of the media as a watchdog. The first lesson to be learned from this experience should be a procedural one: the time to set rules and establish good practices should be ahead, rather than during the crises. To combat disinformation, commitment to a global set of principles and norms by both public authorities and private intermediaries is much needed, in and outside of pandemics. To get there, the first step is to question the view that emergency measures need to come at the expense of democratic guarantees and freedoms. Our search for a global governance approach in relation to the digital ecosystem has never been more urgent.

## References

[bibr1-2056305120948190] AfricaNews. (2020). South Africa enacts regulations criminalizing disinformation on coronavirus outbreak. https://www.africanews.com/2020/03/20/south-africa-enacts-regulations-criminalizing-disinformation-on-coronavirus-outbreak

[bibr2-2056305120948190] Chenou J.-M. Radu R. (2017). The “right to be forgotten”: Negotiating public and private ordering in the European Union. Business & Society, 58(1), 74–102. 10.1177/0007650317717720

[bibr3-2056305120948190] Committee to Protect Journalists. (2020a). 55 Journalists imprisoned between 2016 and 2019 / Charge includes false news. https://cpj.org/data/reports.php?status=Imprisoned&charges%5B%5D=False%20News&start_year=2016&end_year=2019&group_by=location

[bibr4-2056305120948190] Committee to Protect Journalists. (2020b). Bolivia enacts decree criminalizing “disinformation” on COVID-19 outbreak. https://cpj.org/2020/04/bolivia-enacts-decree-criminalizing-disinformation.php

[bibr5-2056305120948190] de Gregorio G. Radu R (2019, 11 24). Counter-disinformation around the world: Comparing state actions [Conference session]. 2019 Global Internet Governance Academic Network Annual Symposium, Berlin, Germany.

[bibr6-2056305120948190] Deuze M. (2007). Media work. Polity Press.

[bibr7-2056305120948190] Facebook. (2020). Community standards enforcement report. https://transparency.facebook.com/community-standards-enforcement

[bibr8-2056305120948190] Goel V. Gettleman J. (2020, 4 2). Under Modi, India’s press is not so free anymore. New York Times. https://www.nytimes.com/2020/04/02/world/asia/modi-india-press-media.html

[bibr9-2056305120948190] International Press Institute. (2020). Tracker on press freedom violations linked to COVID-19 coverage. https://ipi.media/covid19-media-freedom-monitoring/

[bibr10-2056305120948190] Irion K. Radu R. (2013). Delegation to independent regulatory authorities in the media sector: A paradigm shift through the lens of regulatory theory. In: Schulz W. Valcke P. Irion K. (eds.), The independence of the media and its regulatory agencies: Shedding new light on formal and actual independence against the national context (pp. 15–54). Polity Press.

[bibr11-2056305120948190] Media Azi. (2020, 4 10). From 15 days to 45 days. Access to information in Moldova and other countries during the quarantine period. Media Azi. http://www.media-azi.md/ro/stiri/de-la-15-la-45-de-zile-accesul-la-informa%C8%9Bie-%E2%80%9E%C3%AEn-carantin%C4%83%E2%80%9D-%C3%AEn-moldova-%C8%99i-%C3%AEn-alte-state-0

[bibr12-2056305120948190] Polyak G. (2020). “Enabling act” in Hungary: Uncontrolled government power, threatened press. Internet Policy Review. https://policyreview.info/articles/news/enabling-act-hungary-uncontrolled-government-power-threatened-press/1466

[bibr13-2056305120948190] Radu R. (2019). Negotiating internet governance. Oxford University Press.

[bibr14-2056305120948190] Times of India. (2020). Centre seeks SC direction, says no media should publish Covid-19 info before checking facts with government. http://timesofindia.indiatimes.com/articleshow/74918103

[bibr15-2056305120948190] World Health Organization. (2020). Speech by the director-general at the Munich security conference. https://www.who.int/dg/speeches/detail/munich-security-conference

